# Supplementary material for: The Role of Escherichia coli Autotransporters in Urinary Tract Infections and Urosepsis
Source: Int J Mol Sci. 2025 Oct 7;26(19):9760. doi: 10.3390/ijms26199760 (PMC12525524; doi:10.3390/ijms26199760)
Supplement: Supplementary file 1 [file ijms-26-09760-s001.zip › ijms-3873907-supplementary.pdf]

Supplementary Materials

The Role of *Escherichia coli* Autotransporters in Urinary Tract Infections and Urosepsis

Beata Krawczyk [1,*] and Paweł Wityk [2]

[1] Department of Biotechnology and Microbiology, Faculty of Chemistry, Gdańsk University of Technology, 80-233 Gdańsk, Poland
[2] Department of Biopharmaceutics and Pharmacodynamics, Medical University of Gdańsk, 80-210 Gdańsk, Poland; pawel.wityk@gumed.edu.pl
* Correspondence: beata.krawczyk@pg.edu.pl

Table S1 *E. coli* autotransporters in UTI and urosepsis – functional division with evidence type [1-8].

Autotransporter	Evidence type	Notes	Function / Description
Ag43	in vitro/cell-based studies; animal protection / knockout studies; clinical/epidemiological data	In vitro adhesion/biofilm; Murine UTI protection; Prevalence in human UPEC isolates	Self-associating autotransporter; mediates bacterial autoaggregation, biofilm formation, and adhesion to host cells
Tsh	in vitro/cell-based studies; animal protection / knockout studies; clinical/epidemiological data	In vitro adhesion; Avian infection models; Prevalence in poultry and human ExPEC	Temperature-sensitive hemagglutinin; facilitates adhesion to host epithelial cells and colonization in birds and humans
Upa family	in vitro/cell-based studies; animal protection / knockout studies	In vitro biofilm and adhesion; Murine UTI models	Uropathogenic autotransporters; promote adhesion, biofilm formation, and persistence in the urinary tract
SinH	animal protection / knockout studies; clinical/epidemiological data	Murine challenge studies; Prevalence in ST131 pandemic lineage	Novel adhesin autotransporter; implicated in virulence and dissemination of pandemic ExPEC lineages
Vat	in vitro/cell-based studies; animal protection / knockout studies; clinical/epidemiological data	In vitro cytotoxicity; Murine UTI protection; Human isolate prevalence	Vacuolating autotransporter toxin; causes cytotoxic effects in host cells, contributing to urinary tract infections
Sat	in vitro/cell-based studies; animal protection / knockout studies; clinical/epidemiological data	In vitro cytotoxicity/adhesion; Murine protection studies; Prevalence in human isolates	Secreted autotransporter toxin; cytotoxic to epithelial cells and contributes to UTI pathogenesis
Pic	in vitro/cell-based studies; animal protection / knockout studies; clinical/epidemiological data	In vitro immune modulation; Avian infection models; Epidemiologic surveys	Serine protease autotransporter; modulates host immune responses, contributes to intestinal and extraintestinal infections
SPATE general	in vitro/cell-based studies; clinical/epidemiological data	Comparative genomics and prevalence studies; limited in vitro characterization	Serine protease autotransporters of Enterobacteriaceae; diverse roles including cytotoxicity, immune modulation, and colonization

References

1. Vo, J.L.; Martínez Ortiz, G.C.; Subedi, P.; Keerthikumar, S.; Mathivanan, S.; Paxman, J.J.; Heras, B. Autotransporter Adhesins in Escherichia coli Pathogenesis. Proteomics 2017, 17, 1600431. https://doi.org/10.1002/pmic.201600431.
2. Xing, Y.; Clark, J.R.; Chang, J.D.; Chirman, D.M.; Green, S.; Zulk, J.J.; Jelinski, J.; Patras, K.A.; Maresso, A.W. Broad Protective Vaccination against Systemic Escherichia coli with Autotransporter Antigens. PLoS Pathogens 2023, 19, e1011082. https://doi.org/10.1371/journal.ppat.1011082.
3. Ulett, G.C.; Valle, J.; Beloin, C.; Sherlock, O.; Ghigo, J.M.; Schembri, M.A. Functional Analysis of Antigen 43 in Uropathogenic Escherichia coli Reveals A Role in Long-Term Persistence in the Urinary Tract. Infect. Immun. 2007, 75, 3233–3244. https://doi.org/10.1128/IAI.01952-06.
4. Allsopp, L.P.; Totsika, M.; Tree, J.J.; Ulett, G.C.; Mabbett, A.N.; Wells, T.J.; Kobe, B.; Beatson, S.A.; Schembri, M.A. UpaH Is a Newly Identified Autotransporter Protein That Contributes to Biofilm Formation and Bladder Colonization by Uropathogenic Escherichia coli CFT073. Infect. Immun. 2010, 78, 1659–1669. https://doi.org/10.1128/IAI.01010-09.
5. Allsopp, L.P.; Beloin, C.; Moriel, D.G.; Totsika, M.; Ghigo, J.M.; Schembri, M.A. Functional Heterogeneity of the UpaH Autotransporter Protein from Uropathogenic Escherichia coli. J. Bacteriol. 2012, 194, 5769–5782. https://doi.org/10.1128/JB.01264-12.
6. Allsopp, L.P.; Beloin, C.; Ulett, G.C.; Valle, J.; Totsika, M.; Sherlock, O.; Ghigo, J.M.; Schembri, M.A. Molecular Characterization of UpaB and UpaC, Two New Autotransporter Proteins of Uropathogenic Escherichia coli CFT073. Infect. Immun. 2012, 80, 321–332. https://doi.org/10.1128/IAI.05322-11.
7. Wieser, A.; Romann, E.; Magistro, G.; Hoffmann, C.; Nörenberg, D.; Weinert, K.; Schubert, S. A Multiepitope Subunit Vaccine Conveys Protection Against Extraintestinal Pathogenic Escherichia coli in Mice. Infect. Immun. 2010, 78, e00174-10. https://doi.org/10.1128/iai.00174-10.
8. Qiu, L.; Chirman, D.; Clark, J.R.; Xing, Y.; Hernandez Santos, H.; Vaughan, E.E.; Maresso, A.W. Vaccines Against Extraintestinal Pathogenic Escherichia coli (ExPEC): Progress and Challenges. Gut Microbes 2024, 16, 2359691. https://doi.org/10.1080/19490976.2024.2359691.
